# Scalability and Validation of Big Data Bioinformatics Software

**DOI:** 10.1016/j.csbj.2017.07.002

**Published:** 2017-07-20

**Authors:** Andrian Yang, Michael Troup, Joshua W.K. Ho

**Affiliations:** a Victor Chang Cardiac Research Institute, Darlinghurst, NSW 2010, Australia; b St. Vincent's Clinical School, University of New South Wales, Darlinghurst, NSW 2010, Australia

## Abstract

This review examines two important aspects that are central to modern big data bioinformatics analysis – software scalability and validity. We argue that not only are the issues of scalability and validation common to all big data bioinformatics analyses, they can be tackled by conceptually related methodological approaches, namely divide-and-conquer (scalability) and multiple executions (validation). Scalability is defined as the ability for a program to scale based on workload. It has always been an important consideration when developing bioinformatics algorithms and programs. Nonetheless the surge of volume and variety of biological and biomedical data has posed new challenges. We discuss how modern cloud computing and big data programming frameworks such as MapReduce and Spark are being used to effectively implement divide-and-conquer in a distributed computing environment. Validation of software is another important issue in big data bioinformatics that is often ignored. Software validation is the process of determining whether the program under test fulfils the task for which it was designed. Determining the correctness of the computational output of big data bioinformatics software is especially difficult due to the large input space and complex algorithms involved. We discuss how state-of-the-art software testing techniques that are based on the idea of multiple executions, such as metamorphic testing, can be used to implement an effective bioinformatics quality assurance strategy. We hope this review will raise awareness of these critical issues in bioinformatics.

## 1 Introduction

The term big data is used to describe data which are large with respect to the following characteristics: volume (amount of data generated), variety (type of data generated), velocity (speed of data generation), variability (inconsistency of data) and veracity (quality of captured data) [1]. Sequencing data is the most obvious example of big data in the field of bioinformatics, especially with the advancement in next-generation sequencing (NGS) technology and single cell capture technology. Other examples of big data in bioinformatics include electronic health records, which contain a variety of information including phenotypic, diagnostic and treatment information; and medical imaging data, such as those produced by magnetic resonance imaging (MRI), positron emission tomography (PET) and ultrasound. Furthermore, emerging big data relevant to biomedical research also include data from social networks and wearable devices.

One particularly major advancement in experimental molecular biology within the last decade has been the significant increase in sequencing data available for analysis, at a cheaper cost [2]. The cost of sequencing per genome has reduced from $100,000,000 in 2001, to $10,000,000 in 2007, down to a figure close to $1000 today. The $1000 genome is already a reality [3]. Currently, the data that comes out of a NGS machine are in the order of several hundred gigabytes for a single human genome. With the rapid advancement in single-cell capture technology and the increasing interest in single-cell studies, it is expected that the amount of sequencing data generated will increase substantially as each single-cell run can generate profiles for hundreds to thousands of samples [4]. In this review, we will focus specifically on bioinformatics software that deals with NGS data as this is currently one of the most prominent and rapidly expanding source of big data in bioinformatics.

**Fig. 1:**
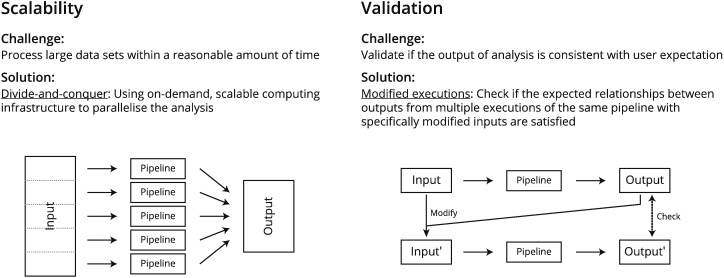
Scalability and validation – two important aspects of big data bioinformatics.

In this review, we argue that the two main issues that are fundamental to designing and running big data bioinformatics analysis are: the need for analysis tools which can scale to handle the large and unpredictable volume of data (Scalability) [4], [5], [6], [7], and methods that can effectively determine whether the output of a big data analysis conforms to the users' expectation (Validation) [8], [9]. In general, there are many other issues associated with bioinformatics big data analysis, such as storage, security and integration [10]. However, these issues have existed even before the rise of big data in bioinformatics, and these issues are typically targeted to specific use cases, such as the storage of sensitive patient data and integration of several specific types of data. Solutions to these specific issues are available [11], [12], though there may be additional challenges associated in implementing the solution due to the increased volume and noise. Nonetheless, these issues are mostly specific to individual application areas. We believe that if we can effectively deal with the scalability and validation problem, it will go a long way in terms of making big data analysis more widespread in practice. This review aims to provide an overview of the technological development that deals with the scalability and validation problems in big data bioinformatics for sequence-based analysis tools (Fig. 1).

## 2 Scalability

Scalability is not a unique challenge in big data analysis. In fact, software scalability has always been an issue since the early days of bioinformatics because of the high algorithmic complexity of some of the algorithms such as those involving global multiple sequence alignment. The early focus on scalability is on parallelising the computation, while a lot less attention is paid on optimally distributing the data. Efforts to make bioinformatics software scalable have continuously been made with the evolution of new hardware technologies, such as cluster computing, grid computing, Graphical Processing Unit (GPU) technology, and cloud computing. Currently in the age of big data bioinformatics, the focus is not only on parallelising computational intensive algorithms, but also on highly distributed storage and efficient communication among various distributed storage or computational units. Furthermore, the volume and variety of data can change dynamically in response to potentially unpredictable user demand. For example, in a medium-sized local sequencing centre, the volume of data can grow rapidly during certain unexpected peak periods, but remain constant during other periods. This variability of demand on computational resources is also a critical feature of modern big data bioinformatics analysis. In this section, we will review the evolution of parallel distributed computing technologies and how they have contributed to solving the issue of scalability of bioinformatics software. In particular, we will discuss how modern cloud computing technology and big data analysis frameworks, such as MapReduce and Spark, can be effectively used to deal with the scalability problem in the big data era.

### 2.1 Cluster Computing

Early attempts at scaling bioinformatics software beyond massively parallel (super) computers involved networking individual computers into clusters to form a parallelised distributed-memory machine. In this configuration, computations are performed by splitting and distributing tasks across Central Processing Units (CPUs) in a way that is similar to the symmetric multiprocessing (SMP) approach utilised in massively parallel computers. Unlike SMP, which relies on a shared main memory, clusters have distributed-memory, with each node having its own memory and hard drive, thus presenting a new challenge in developing software for cluster environments. To help with the development of cluster-based software, communications protocols and software tools, such as Message Passing Interface (MPI) [13] and Parallel Virtual Machine (PVM) [14], have been developed for orchestrating computations across nodes. An example of bioinformatics software designed for cluster computing is mpiBLAST, an MPI-based, parallelised implementation of the basic local alignment search tool (BLAST) algorithm which performs pairwise sequence similarity between a query sequence and a library or database of sequences [15]. The approach taken by mpiBLAST includes the use of a distributed database to reduce both the number of sequences searched and disk I/O in each node, thereby improving the performance of the BLAST algorithm. MASON is another example of MPI-based bioinformatics software for performing multiple sequence alignment algorithms using the ClustalW algorithm [16]. MASON speeds up the execution of ClustalW by parallelising the time- and compute-intensive step of calculating a distance matrix of the input sequences, and the final progressive alignment stage.

### 2.2 Grid Computing

The next approach in scaling bioinformatics software comes with the introduction of grid computing, which represents an evolution in the distributed computing infrastructure. Grid computing allows for a collection of heterogeneous hardware, such as desktops, servers and clusters, which may be located in different geographical locations, to be connected through the Internet to form a massively distributed high performance environment [17]. Although conceptually similar to a cluster, grid computing presents a different set of challenges for developing software. The comparatively large latency between nodes in a grid environment compared to a cluster environment means that software for grid needs to be designed with minimum communication between nodes. Furthermore, the heterogeneity of the grid environment means that software may need to take into account differences in the underlying operating system and the system architecture of the nodes. Development of bioinformatics software for a Grid typically uses a middleware layer which abstracts away the underlying grid architecture management. A widely-used middleware layer is the Globus Toolkit, a software toolkit for managing and developing in a grid environment [18]. An example of a bioinformatics software for the Grid environment is GridBLAST, an implementation of BLAST with Globus as the middleware layer for distributing BLAST queries across nodes in the grid [19]. Aside from Globus, there are also bioinformatics-specific grid middleware layers such as myGrid [20] and Squid [21].

### 2.3 GPGPU

The introduction of general-purpose computing on GPUs (GPGPUs) revived interest in the massively parallel approach initially used before the distributed computing approach became the mainstream. GPUs are specialised processing units designed for performing graphic rendering. Unlike a CPU, which has a limited number of multi-processing units, a GPU has a large number of processing units in the order of hundreds and thousands, thus allowing for the high computational throughput required for rendering 3D graphics. Though the GPU is not a new technology, early GPU architectures were hardwired for graphics rendering and thus it was not until the development of a more generalised architecture which supported general-purpose computing that GPU become more widely used for computation. As with other technologies, there are challenges associated with implementing bioinformatics software on GPUs due to the single instruction multiple data (SIMD) programming paradigm where data are processed in parallel using the same set of instructions. Due to its architecture, computation for GPU will need to be designed with minimum level of branching (homogenous execution) with high computational complexity in order to fully take advantage of the high multiprocessing capability of the GPU. One of the early bioinformatics software utilising GPGPUs is GPU-RAxML (Randomized Axelerated Maximum Likelihood), a GPU based implementation of RAxML program for the construction of phylogenetic trees using a Maximum Likelihood method [22]. GPU-RAxML utilises the BrookGPU programming environment [23], which supports both OpenGL and DirectX graphic libraries, to parallelise the longest loop in the RAxML program, which accounts for 50% of the execution time. Another example of GPU-accelerated bioinformatics software is CUDASW ++, an implementation of the dynamic-programming based Smith-Waterman (SW) algorithm for local sequence alignment [24]. CUDASW ++ utilises the CUDA (Compute Unified Device Architecture) programming environment [25], developed for NVIDIA GPU, to implement two parallelisation strategies of the SW algorithm based on the length of the subject sequence.

### 2.4 Cloud Computing

Cloud computing is defined by the United States' National Institute of Standards and Technology as ‘…a model for enabling ubiquitous, convenient, on-demand network access to a shared pool of configurable computing resources (e.g., networks, servers, storage, applications and services) that can be rapidly provisioned and released with minimal management effort or service provider interaction.’ [26]. Though similar to cluster and grid computing in that cloud computing is based on collections of commodity hardware, cloud computing utilises hypervisor technology to provide dynamic access to ‘virtualised’ computing resources. Virtualisation enables a single hardware resource to ‘host’ a number of independent virtual machines that can run on different operating systems, and which each share some of the underlying hardware resources. Cloud computing is very well suited for big data bioinformatics applications as it allows for on-demand provisioning of resources with a pay-as-you-go model, thus eliminating the need of purchasing and maintaining costly local computing infrastructure for performing analyses. Furthermore, the on-demand provisioning of cloud computing also enables the scaling of computational resources to match the workload being performed at any particular time.

Modern cloud computing is widely accessible, and is not limited to researchers in a large university or institution that have a large computer cluster. There are currently three major cloud computing providers which offer pay-as-you-go access to computing resources – Amazon Web Services (AWS), Google Cloud Platform and Microsoft Azure. There are also a number of cloud computing platforms which are freely available to researchers, such as Atmosphere cloud from CyVerse [27], EGI cloud compute [28] and Nectar Research Cloud [29]. Cloud providers can offer access to “instances”, which are the virtual machines for which a user can select from various configurations, including number of CPU, amount of RAM and operating system. The configuration can range from that of a notebook (1 CPU, 2 GB RAM), to a desktop workstation (8 CPU, 16 GB RAM) or even a supercomputer (128 CPU, 2 TB RAM). Furthermore, some cloud providers also offer specialised instances, such as those with a field-programmable gate array (FPGA) or GPUs. An instance typically starts within minutes, and the user is charged for the duration of the instance's lifetime. As well as traditional instances (servers or workstations in non-cloud terminology), cloud providers offer access to a range of other software and hardware offerings. Other offerings include compute facilities, storage and content delivery, database, networking, analytics, enterprise applications, mobile services, developer tools, management and security tools, and application services.

The services offered by cloud computing providers can be roughly categorised into Data as a Service (DaaS), Software as a Service (SaaS), Platform as a Service (PaaS), and Infrastructure as a Service (IaaS). Data as a Service is a service where data is provided on-demand for users through the Internet. This type of service is particularly relevant for bioinformatics with the increasing production of biological data as a way to store and share data for analysis. There are currently two DaaS providers for biological data – AWS Public Dataset [30] and Google Genomics Public Data [31] – which provide free access to public data sources such as various reference genomes, the 1000 genomes project and The Cancer Genome Atlas. Software as a Service, on the other hand, provides on-demand access to software without the need to manage the underlying hardware and software resources. There have been many bioinformatics SaaS solutions to perform tasks ranging from short-read alignment (CloudAligner [32] and SparkBWA [33]), variant calling (Halvade [34] and Churchill [35]) and RNA-seq analysis (Oqtans [36] and Falco [37]). In Platform as a Service, users are provided with a platform for developing their own software. Some PaaS providers also provide support for automatically scaling the computing resource used based on the workload being run. Unlike the SaaS model where the user is only able to access the provided software, PaaS allows bioinformaticians to develop their own custom bioinformatics pipeline. Examples of PaaS platforms in bioinformatics include Galaxy Cloud [38], a cloud-based scientific workflow system, and DNANexus [39], an AWS based analysis and management platform for next-generation sequencing data. Finally, Infrastructure as a Service is the most basic type of service where users are given access to the virtualised ‘instance’. In bioinformatics, IaaS solutions are typically virtual machine (VM) containers in which a user can deploy to their own customised instance on the cloud. Both CloudBioLinux [40] and CloVR [41] are examples of IaaS solutions for performing bioinformatics analysis. Some cloud providers, especially those targeted for researchers, also provide some pre-built VM snapshots or images with pre-configured software tools which can be deployed to the instance for ease of use.

Containerisation is another virtualisation approach which is becoming increasingly popular in bioinformatics - driven by the introduction of Docker, a simplified, cross-platform tool for deploying application software to containers. Unlike virtual machines, container-based virtualisation - also known as operating-system level of virtualisation - only creates lightweight, isolated virtual environments (called containers) which utilise the system kernel without virtualising the underlying hardware. Containers have high degree of portability as they provide a consistent environment for software regardless of where it is executed. This is particularly useful in the bioinformatics field to help researchers in reproducing their studies by setting up ‘analysis’ containers which can be reused and shared [42]. Due to its popularity in the software industry, there are a growing number of cloud computing providers which support the deployment of containers, such as AWS EC2 container service, Google Container Engine and Azure Container service.

### 2.5 Programming Frameworks for Big Data Analysis on the Cloud

**Fig. 2:**
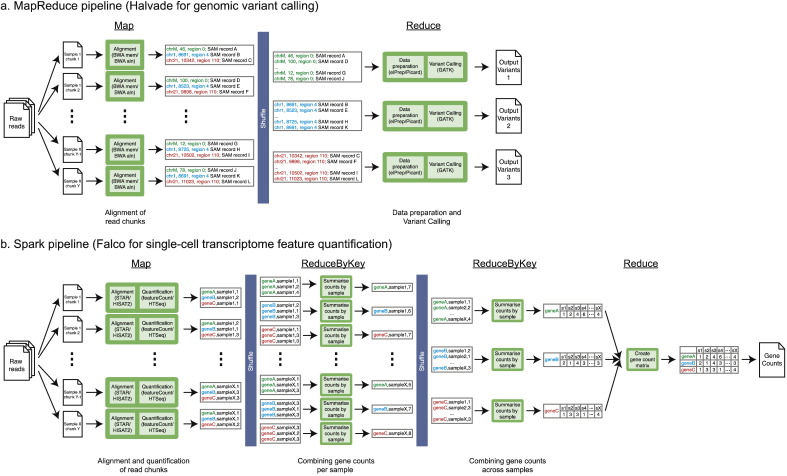
Examples of MapReduce-based (a) and Spark-based (b) big data bioinformatics analysis frameworks.

One important factor that has contributed to the widespread adoption of cloud computing is the development of software frameworks for big data analysis. The nature of big data means that it is difficult to analyse them efficiently using existing computational and statistical methods since they often do not deal with distributed storage and often do not cope well with large data size. MapReduce was introduced by Google in 2004 as both a programming model and an implementation for performing parallelised and distributed big data analyses on large clusters of commodity hardware [43]. In the MapReduce programming model, computation is expressed as a series of Map and Reduce steps, which consumes and produces a list of key-value pairs. Apache Hadoop is an open-source implementation of the MapReduce programming model. The Hadoop framework is composed of several modules, including the Hadoop Distributed File System (HDFS), which is a distributed and fault-tolerant storage system, Hadoop YARN for resource management and job scheduling/monitoring, and the Hadoop MapReduce engine for analysis of data. Halvade is an example of a Hadoop-based bioinformatics tool for performing read alignment and variant calling for genomic data (Fig. 2a). The Halvade framework is composed of a Map step, which performs alignment of sequencing reads to the reference genome using Burrows-Wheeler Aligner (BWA), and a Reduce step, which performs variant calling in a chromosomal region using Genome Analysis Toolkit (GATK). Other examples of MapReduce-based bioinformatics analysis tools include Myrna, which performs RNA-sequencing gene expression analysis [44] and CloudAligner, a short-read sequence aligner.

One disadvantage of the MapReduce programming model is the need to decompose tasks into a series of map and reduce steps. Iterative tasks, such as clustering, do not suit the MapReduce model and thus perform poorly when implemented as MapReduce tasks. Apache Spark is a general purpose engine for big data analysis designed to support tasks incompatible with the MapReduce model, such as iterative tasks and streaming analytics, through the use of in-memory computation [45]. Unlike the MapReduce model, Spark provides a variety of operations, namely transformations and actions, which can be chained to form a complex workflow. Furthermore, Spark supports multiple deployment modes – local mode, standalone cluster mode and using cluster managers, such as YARN – thus allowing for integration with the Hadoop framework. An example of a Spark-based tool is Falco, a single-cell RNA-sequencing processing framework for feature quantification [37]. The Falco pipeline utilises Spark in the main analysis step for performing alignment and quantification of reads to produce gene counts from a portion of the sample, which are then combined to obtain the total gene counts per sample (Fig. 2b). VariantSpark [46] and SparkBWA [33] are other examples of Spark-based tools for performing population-scale clustering based on genomic variation, and read alignment of genomic data, respectively.

## 3 Validation

Another very important, yet largely ignored, area of big data bioinformatics is the problem of software validation – a process that determines whether the output of a software program conforms to users' expectations of the intended function. For example, NGS-based assays, such as whole genome sequencing (WGS), are now ubiquitous in almost all areas of biomedical research, and is beginning to be used in translational applications, as exemplified by the use of NGS-based targeted gene panels in clinical pathology laboratories [47]. Ensuring that the computational pipeline is producing the correct and valid results is critical, particularly in a clinical setting. Many bioinformatics analysis programs have been developed to analyse NGS data, however recent studies found the results generated by different bioinformatics pipelines based on the same sequencing data can differ substantially. Bennett et al. [48] recently reported a study initiated by the Boston Children's Hospital. Twenty three international bioinformatics groups analysed NGS data relating to a group of participants with known genomic disorders. Only one third of the groups reported any of the known variants that the participants were known to contain. In another study, O'Rawe et al. [49] reported on the lack of agreement between a number of variant calling pipelines when analysing the same data. Beyond variant calling, low concordance is also observed when comparing analysis programs used in RNA-seq analysis [9], ChIP-seq analysis [50], [51], [52], and BS-seq analysis [53]. These troubling reports highlight the urgent need to ensure bioinformatics pipelines for NGS analysis are subjected to better validation, especially for translational genomic medicine applications.

Lack of quality assurance (QA) of software pipelines is a critical problem that has a widespread impact on all areas of biomedical research and clinical translation of NGS-based assays. The main difficulty is that while it is possible to perform validation of a software program in a test environment using simulation data or a limited number of ‘gold standard’ test data sets, it is generally impossible to determine the correctness of the software program on real data within the software's deployment environment. Many bioinformatics programs have a large and complex input space, implying a large number of different test cases need to be sampled to ensure the input space is comprehensively covered. This implies that the testing results on a suite of simulation data may or may not fully recapitulate the characteristics of the real data. Obviously it would be the best to directly validate the correctness of a program using specific real input data, but it is often difficult, if not impossible, to decide if the output based on this real data is correct – otherwise the program would not be needed in the first place. This type of user-oriented software QA is lacking in bioinformatics, especially in NGS-based data analysis.

### 3.1 Current Bioinformatics Software Testing Approaches Used in the Genomics Field

Using a variant calling pipeline for analysing WGS data as an example, a common approach to testing a variant calling pipeline (involving short read alignment, variant calling, and variant annotation) to is to run the pipeline using a gold standard reference test data and compare the results (*i.e.*, the variants called) with the expected reference output. In the US, The National Institute of Standards and Technology (NIST), along with the Genome in a Bottle Consortium [54] and the Food and Drug Administration (FDA), is developing such reference material for whole human genomes. One of the goals of this partnership is to develop reference standards, methods and data for whole human genome sequencing. Reference data sets can also be obtained from the manufacturer of the particular sequencing machine – *e.g.*, Illumina's *The BaseSpace Platinum Genomes Project* and the *Variant Calling Assessment Tool* (VCAT) [55]. While this approach is a good starting point, it only verifies a very limited number of input/output combinations. All humans are different, and clinicians want to have confidence that the pipeline will work for every new case. For testing partial results, a smaller FASTQ input file can be used to either manually or automatically determine if the output is correct, or *Sanger* sequencing [56] can be used to verify a subset of the data.

There are a number of simulation packages available that can simulate read data, and provide a ‘ground truth’ VCF file for the expected output. Two such programs are ART [57], and VarSim [58]. Such programs allow for the generation of large amounts of data to be tested. While partially addressing the ‘gold-standard’ issue of lack of data, there are additional sources of uncertainty introduced depending upon the simulation process used.

As O'Rawe et al. [49] demonstrated, and as also commented on by Li et al. [59], another popular testing method in the genomic sequencing bioinformatics area is that of *N-version programming*. Multiple versions of independently developed programs that are meant to solve the same problem are executed, and the outputs of these programs are compared to check if they are same. One obvious shortcoming of this is that if different results are obtained, the tester may not be able to determine which program is correct.

In this review, we argue that adapting and extending state-of-the-art methods in the field of software testing can provide a foundation for developing evidence-based, effective and readily deployable strategies for bioinformatics QA.

### 3.2 Software Testing Concepts and Approaches

According to Myers et al. [60], the goal of software testing in general is to ensure that a program works as intended or, perhaps more realistically, to identify as many errors as possible in the underlying software. There are testing techniques that identify problems by exploring the internal structure of the software, applying inputs based on the various logic built into the system. Such a type of testing is termed *white-box* testing. *Black-box* testing, on the other hand, is the process of examining the output or behaviour of a software system, without any examination of the internal workings of the system. In many practical situations, bioinformatics pipelines are comprised of many individually complex programs solving some subset of an overall task. Some of the individual components may include software for which the source code is not publicly available. Therefore, we argue that in many cases, a black-box testing approach is the only practical option.

In many classes of software applications, it may be possible to verify the correctness of a small subset of output, but it is often impossible to obtain a systematic mechanism to verify all the output. The mechanism that enables any input to be verified is called an *oracle* [61]. In most practical cases, bioinformatics big data analysis software lacks an oracle [62]. Ideally, we would like to directly test the correctness of a bioinformatics program with respect to a real input data set. Nonetheless, in the absence of an oracle, it is impossible to easily determine the correctness of a program with respect to an input.

### 3.3 Metamorphic Testing

*Metamorphic testing* (MT) is a state-of-the-art technique of software testing, first introduced by Chen et al. in 1998 [63], as a method to deal with situations where there is no oracle. Rather than verifying individual output values, multiple related input test data are executed by the same program under test, and their corresponding outputs are examined to determine if known relationships hold. Each particular domain-specific relationship is termed a *metamorphic relation* (MR). Some test cases (namely *source test cases* in the context of MT) can be selected by random or based on real data. Further test cases (namely *follow-up test cases* in the context of MT) can be generated based on the source test cases and according to the MRs. All test cases are executed, and then the outputs of the source and follow-up test cases are checked against the MRs. If any pair of source and follow-up test cases violates (that is, does not satisfy) their corresponding MR, the tester can say that a failure is detected and hence conclude that the program has bugs. In other words, MT tests for properties that users expect of a correct program.

**Fig. 3:**
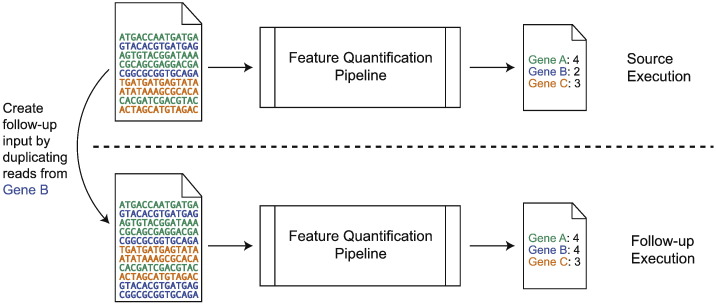
An example to illustrate how metamorphic testing (MT) can be used to test the correctness of a RNA-seq feature quantification pipeline.

As an example, here is an MR that can be used to test the correctness of a RNA-seq gene expression feature quantification pipeline (Fig. 3): After the source execution of the feature quantification pipeline using real data, we select a gene (*e.g.*, Gene B in Fig. 3) with non-zero read count, and duplicate all the reads that are mapped to this gene to construct the input for the follow-up test case. The expectation is that if Gene B has read count of *x* in the source test case, the read count of *x* in the follow-up test case should be 2*x*, and the read count of all other genes should remain identical to that of the source case. This rather simple example illustrates two important features of MT: (1) we can conduct the test with real data, (2) the follow-up test case is constructed by both the input and output of the source execution, and (3) it is possible to generate additional follow-up test cases using this same MR by targeting different genes.

Let's consider another example to illustrate how MT can be applied to test a supervised machine learning classifier. Let's denote classifier *C* takes three sets of input: training data set *D*_train_, labels of the training data set *L*_train_, and the test sample *d*_test_. The goal of the classifier *C* is to predict the label of *d*_test_, *i.e.*, *l*_test_ = *C* (*D*_train_, *L*_train_, *d*_test_). It is in general very difficult to validate the correctness of any given output prediction *l*_test_, since if we can easily determine the correctness of the output, there is no need to build the classifier *C* in the first place. In the field of machine learning, validation is normally performed by cross-validation or by the use of a held-out test set. Nonetheless, these strategies rely on using data with known labels. They are not able to directly test the validity of the specific prediction output *l*_test_ based on the input *d*_test_. The most critical step in MT is to identify some desirable properties that *C* should satisfy, and use them to construct MRs. Here are several simple MRs for illustration purposes (a more comprehensive list of MRs can be found in Xie et al. [64]):

• **MR1 (consistency upon adding an uninformative feature)**: After executing the source test case, add one additional feature with the same value, such as 0, to all samples in *D*_train_ and *d*_test_. Since this feature does not discriminate between samples from different classes, this feature can be treated as an uninformative feature. We expect that the output of the follow-up test case is the same as that of the source test case.
• **MR2 (consistency upon re-prediction)**: After executing the source test case, append *d*_test_ to *D*_train_, and *l*_test_ to *L*_train_. We use the modified training data to predict the label for *d*_test_ in the follow-up test case. We expect that the output of the follow-up test case is the same as that of the source test case.

It should be noted that these MRs were specified without knowing the classification algorithm (*k* Nearest Neighbour classifier, Support Vector Machine, neural network, *etc.*). They were derived from some intended user behaviours of a reasonable supervised classifier. Some of these MRs may be necessary properties of a specific supervised classification algorithm if these properties can be derived from the specification of the algorithm. In this case, MT is said to be performing software verification. Otherwise, MRs that are derived primarily from user expectation is said to be performing software validation. Violation of one or more MR indicates potential limitations in the algorithm or defects in the software implementation. Even though passing all MRs does not necessarily imply the correctness of the program under test, it does provide confidence that the program is behaving as expected with respect to your specific input data. Recent study showed that a small number of diverse MRs, even those identified in an *ad hoc* manner, had a similar fault-detection capability to a true test oracle [65]. Importantly, MT can use **real data** as source test cases, therefore allowing testers to test the correctness of the program with respect to the specific real data in its normal operational environment. This unique feature of MT is highly desirable for bioinformatics QA.

MT has been used in the testing of many types of software, such as web services [66], compilers [67], feature models [68], machine learning [64], partial differential equations [69], and bioinformatics [70]. Within the field of bioinformatics, there have been a number of studies that establish the case for MT. In 2009, Chen et al. [71] apply MT to two different bioinformatics problems: network simulation and, short sequence mapping. The authors show that MT can be simple to apply, has the potential to be automated, and is applicable to a diverse range of programs. A key point of interest to bioinformaticians, is that the construction of relevant MRs draws on domain knowledge. MT also has its limitations, and just because all MR's are satisfied does not imply that the program is free of defects – a fundamental limitation of all dynamic software testing techniques.

## 4 Discussion

This review has surveyed some methods for implementing scalability and validation for big data bioinformatics software, more specifically for sequence-based analysis tools. With the increasing push to generate sequencing data sets at the single-cell level of resolution [72], [73], it is clear that analysis of these large amount of NGS data will need to be performed on large-scale cloud and/or grid infrastructure, using software which can efficiently scale to handle the large amount of data. However, the techniques and concepts introduced in the review are not limited to sequence-based analysis tools and can be adopted to bioinformatics software in other fields. There are already a number of tools which have been developed using big data frameworks in other bioinformatics fields, such as AutoDockCloud, a tool for drug discovery through virtual molecular docking which utilises the Hadoop MapReduce framework [74], and PeakRanger, a tool for calling peaks from chromatin immunoprecipitation sequencing (ChIP-Seq) data also on the MapReduce framework [75].

The central methodological concept for dealing with scalability is divide-and-conquer. The goal is to divide a large task into many small portions and process them in parallel. The main requirement for the success of such an approach is to have an efficient means to manage the division and merging of data and computation, and the availability of a variable amount of IT resources on demand. Modern cloud-based computing technology and big data programming frameworks provide a systematic means to tackle these issues.

The central methodological concept for dealing with validation is multiple executions. Through executing a program multiple times on related inputs (based on real input or simulated data) we are able to check whether the output conforms to a set of properties that a user might expect of the correct results. The idea is that specifically designed multiple executions allows a user to extract useful information about the program, and whether the output is likely valid with respect to users' expectations.

Methodologically, the idea of multiple executions involves making the computational task larger, which further highlights the importance of scalability. Without a scalable computing solution, it would be difficult to effectively implement a validation solution in practice. As an illustration of this concept, our team has recently developed a pilot MT-based validation framework for a whole exome sequencing processing pipeline, which is easy, cost effective, and available as an on-demand platform on the cloud [70]. We showed that using this cloud-based MT validation framework reduced the overall runtime seven-fold compared to running it on a standalone computer with comparable power. This initial effort on using cloud-based computing to substantially speed up the validation runtime highlights the relationship between scalability and validation. We believe further research in this area will contribute toward building scalable and valid bioinformatics programs for big biological data.
